# Prevalence of Dental Caries in relation to Body Mass Index, Daily Sugar Intake, and Oral Hygiene Status in 12-Year-Old School Children in Mathura City: A Pilot Study

**DOI:** 10.1155/2014/921823

**Published:** 2014-02-12

**Authors:** Prahlad Gupta, Nidhi Gupta, Harkanwal Preet Singh

**Affiliations:** ^1^Department of Public Health Dentistry, Dasmesh Institute of Research and Dental Sciences, Faridkot, Punjab 151203, India; ^2^Department of Prosthodontics, Dasmesh Institute of Research and Dental Sciences, Faridkot, Punjab 151203, India; ^3^Department of Oral Pathology and Microbiology, Dasmesh Institute of Research and Dental Sciences, Faridkot, Punjab 151203, India

## Abstract

*Aim*. To correlate the prevalence of dental caries to body mass index, daily sugar intake, and oral hygiene status of 12-year-old school children of Mathura city. *Material and Methods*. The study design was cross-sectional and included 100 school children aged 12 years (*n* = 50 boys and *n* = 50 girls) who were randomly selected from two schools based upon inclusion and exclusion criteria. Body weight/height was recorded and BMI was calculated and plotted on CDC-BMI for age growth charts/curves for boys and girls to obtain percentile ranking. Dental caries was recorded using WHO criteria. Oral hygiene status of the study subjects was assessed using oral hygiene index-simplified. Data regarding the daily sugar intake was recorded using 24-hour recall diet frequency chart. The data obtained was analysed using SPSS version 11.5 for windows. *Result*. Only 27 subjects were affected by caries. The mean DMFT/dmft was 0.37 ± 0.79 and 0.12 ± 0.60, respectively. Statistical analysis by means of a logistic regression model revealed that only oral hygiene status had a significant effect on caries prevalence (OR = 5.061, *P* = 0.004), whereas daily sugar intake and body mass index had no significant effect. *Conclusion*. From the analysis, it was concluded that oral hygiene status had a significant effect on caries prevalence of 12-year-old school children of Mathura city.

## 1. Introduction

Dental Caries is a chronic disease which can affect us at any age. If untreated, it can lead to pain and discomfort and finally loss of teeth. Caries is one of the most common diseases of childhood. The disease is not self-limiting and without adequate intervention, the process can continue until the tooth is destroyed. The term “caries” denotes both the disease process and its consequences, that is, the damage caused by the disease process [1]. The World Health Organization's report on oral health in 2003 and Global Oral Data Bank of WHO confirm the international distribution of dental caries and stated that by the age of 12 only 15 to 30% of the population were caries-free with a global DMFT of 1.74 [2–4]. The global distribution of dental caries presents a varied picture; countries with low caries prevalence are experiencing an unprecedented increase in caries prevalence and severity of dental caries. On the other hand, in developed countries a reduction of dental caries incidence and improvement of gingival health care are evident. This decline in dental caries was mainly due to appropriate use of fluorides and preventive oral health care measures. The scenario in India is not different from developing countries [5].

Dental caries has a multifactorial aetiology in which there is interplay of three principal factors: the host (saliva and teeth), the microflora (plaque), and the substrate (diet) and a fourth factor: time. There is no single test that takes into consideration all these factors and can accurately predict an individual's susceptibility to caries. The risk of dental caries can be evaluated by analysing and integrating several causative factors such as fluoride, microbial plaque, diet, bacterial and salivary activity, and social and life style related behavioural factors [1].

Excessive body weight in children is a major public health problem. According to National Family Health Survey (NFHS), obesity has reached epidemic proportions in India, affecting 5% of the country's population. India is following a trend of other developing countries that are steadily becoming more obese [6, 7]. Obesity status in children is measured by assessment of body mass index (BMI) corresponding to gender and age [6]. Consumption of soft drinks and fast foods together with less activity and exercise contributed to the increasing number of overweight people worldwide [8]. High sugar intake, for example, sugar containing snacks and soft drinks, is reported to be more common among overweight and obese children/adolescents than those with normal weight. Frequent sugar intake is also a recognized risk factor for dental caries. Thus, the eating pattern among overweight or obese children may be a common risk factor in overweight children and dental caries [9]. Given that the strong evidence supporting the relation between dental caries with indiscriminate dietary intake has been linked to the development of obesity at a young age, a link between dental caries and weight is biologically possible [6]. The role of sugar (and other fermentable carbohydrates such as highly refined flour) as a risk factor in the initiation and progression of dental caries is overwhelming. Sugar acts as a favoured substrate for the cariogenic bacteria that reside in dental plaque, particularly the mutans streptococci, and the acid by-products of this metabolic process induce demineralization of the enamel surface. Whether this initial demineralization proceeds to clinically detectable caries or whether the lesion is remineralized by plaque minerals depends on a number of factors, of which the amount and frequency of further sugars consumption are of utmost importance [10]. Another risk factor for development of caries is the existence of bacterial plaque on the teeth. Caries can be reduced by mechanical removal of plaque from tooth surfaces; however, most children do not remove it effectively which means the deficiency of maintenance of good oral hygiene. Several studies have shown that, in countries where proper oral hygiene is followed, caries prevalence has decreased despite increases in sugar consumption, thus marking the importance of oral hygiene in caries etiology [11, 12].

Dental caries is a multifactorial disorder and it is difficult to assess all the associated risk factors simultaneously. There have been no studies documented in literature in this part of India assessing the prevalence of dental caries in relation to body mass index, daily sugar intake, and oral hygiene. So an attempt was made to assess the prevalence of dental caries in relation to body mass index, daily sugar intake, and oral hygiene status in 12-year-old school children in Mathura city. Cross-sectional study was designed to evaluate the daily sugar intake, oral hygiene status, and body mass index and to correlate each of them with the prevalence of dental caries among 12-year-old school children in Mathura city.

## 2. Material and Method

Ethical permission from institutions ethical committee was taken before the commencement of study. Consent from subjects, parents, and school was also taken.

Sample size was determined by the formula based on the study population:

(1) $$\begin{array}{c} n=\frac{4pqn}{e^{2}\left(N-1\right)+4\;pq}, \end{array}$$

where *p* = prevalence 27% (prevalence of dental caries obtained from previous study), *q* = (1 − *p*) = 100 − 27 = 73, *e* = permissible error in estimation of prevalence 10%, *N* = study population 5000 (Department of Education Mathura), and *n* = sample size.

The estimated sample size for the study based on the prevalence of the dental caries came out to be 860.10% of sample size; that is, 86 was included in pilot study, but to avoid any error slightly higher sample of 100 was taken for study.

This study was planned to be conducted in high schools of Mathura city in 12-year-old children. There are 141 primary schools and 37 high schools as per the record in the District Education Departments of Mathura. Out of 37 high schools, 16 are government aided and the rest are all private institutions. Government institutions had children with similar socioeconomic and cultural background. Children do not have any specific habit such as tobacco chewing and smoking, but they were very fond of having sugar candies.

Level of fluoride ion concentration in drinking water in Mathura city is in optimum range and subjects maintained their oral hygiene by using fluoridated toothpaste.

Out of 16 government aided high schools, 2 schools were randomly selected to obtain the sample size of 100 study subjects having similar socioeconomic and cultural background. Subjects who were willing to participate, have completed 12 years of age, and were continuously residing in Mathura city right from their birth were included in the study whereas subjects who were suffering from any acute or chronic diseases and were under medication, were below 12 years of age and above 13 years of age, and did not obtain parental consent were excluded from the study. A proforma was used for collection of data in the study.

### 2.1. Anthropometric Measurements

Body weight of study subjects was measured using standardized digital weighing machine. The fractional weight below 500 grams and above 500 grams was rounded to the nearest whole number. Height of study subjects was measured using a measuring tape and recorded in meters. Measurement of weight and height was taken without shoes and with their school dress. From the above data, BMI was calculated and plotted on CDC-BMI for age growth charts/curves for boys and girls to obtain a percentile ranking and subjects were categorized as follows [13].

 Underweight: less than 5th percentile.
 Healthy weight: 5th percentile to less than 85th percentile.
 At risk of overweight: 85th to less than the 95th percentile.
 Overweight: equal to or greater than the 95th percentile.

### 2.2. Dental Caries

Dental caries status was collected using Dentition Status of WHO criteria mentioned in Basic Oral Health Survey Methodology (1997) [14] and from the above data DMFT/dmft was calculated.

### 2.3. Oral Hygiene Status

Oral hygiene of study subjects was determined using oral hygiene index-simplified (OHI-S) by Greene and Vermilion [15]. This index is based upon two parameters: Debris and Calculus and it has been validated by other authors in 12-year-old children of different geographic region.

### 2.4. Daily Sugar Intake

Data regarding the daily sugar intake was recorded using 24-hour recall diet frequency chart and the subjects were grouped into excellent, good, and watch out zone based upon sugar sweet score (see Table 1) [16].

All examinations and data collection were done by a single examiner and proforma was filled by a recording assistant after standardization. The examination of study subjects was carried out in their school premises using natural light, ordinary chair, plain mouth mirror and CPI probe for dental caries, and explorer no. 5 (Shepard's hook) for OHI-S. Presterilized armamentarium was used to carry out the examinations.

### 2.5. Statistical Analysis

The data obtained was analysed using SPSS version 11.5 for windows. Mean and standard deviations were calculated for each clinical parameter. Differences between means were tested with one-way ANOVA followed by post hoc tukey's test. Independent effects of BMI, oral hygiene status, and daily sugar intake on caries prevalence were tested using linear multiple regression analysis. Significance for all statistical tests was predetermined at a probability (*P*) value of 0.05 or less.

## 3. Results

An epidemiological survey conducted showed that the study population consisted of 100 school children, out of which 50 (50%) were males and 50 (50%) were females.  Table 2 shows sex-wise distribution of various characteristics of study population collected by survey. One-way ANOVA was applied to determine the association between mean DMFT and BMI categories of study population (underweight, health weight, at risk of overweight and overweight) but no significant association was found (*F* = 1.145, *P* = 0.335, N.S.) but when it was applied to determine the association between mean dmft and BMI Categories of study population (underweight, health weight, at risk of overweight and overweight), significant association was found (*F* = 7.783, *P* = 0.000, S). Similarly, one-way ANOVA was applied to determine the association between mean DMFT/dmft and daily sugar intake categories of study population (excellent, good, and watch out); no significant associations were found (*F* = 1.348, *P* = 0.265, N.S., and *F* = 0.489, *P* = 0.615, N.S.), respectively. When one-way ANOVA was applied to determine the association between mean DMFT/dmft and oral hygiene status of study population (good, fair, and poor), no significant associations were found (*F* = 2.563, *P* = 0.082, N.S., and *F* = 1.051, *P* = 0.354, N.S.). Multiple linear regression analysis was done to determine the independent effects of BMI, oral hygiene status, and daily sugar intake on caries prevalence. It was found that oral hygiene status had a significant effect on caries prevalence (OR = 5.061, *P* = 0.004, S). However, body mass index and daily sugar intake had no significant effect on caries prevalence (Table 3).

## 4. Discussion

The main objective of the present study was to determine the prevalence of dental caries in relation to body mass index, daily sugar intake, and oral hygiene status of 12-year-old school children of the Mathura city. Our study found a low caries prevalence (27%) with a mean DMFT of 0.37 and mean dmft of 0.12, respectively, when compared with the global DMFT for 12-year-olds [4]. Similar results were obtained in a study by David et al. (2005) [17] who reported 27% prevalence of dental caries with a mean DMFT of 0.5. Our study found no statistically significant association between DMFT and BMI (*F* = 1.145, *P* = 0.335, N.S.). Similarly, Tramini et al. [18] found no significant association between DMFT and BMI. This finding is consistent with the results from the prospective study by Pinto et al. [6], where no correlation between dental decay and BMI was detected in a multiple regression analysis. Kopycka-Kedzierawski et al. [9] even found an inverse association between BMI and caries experience: overweight children were less likely to have caries experience than normal weight children aged 6–11 years. After having performed a systematic review of obesity and dental caries, Kantovitz et al. [19] concluded that only one study with high level of evidence showed direct association between obesity and dental caries.

Recent systematic review and meta-analysis conducted by Hayedn et al. [20] showed that, overall, there was a significant relationship between childhood obesity and dental caries. However, this relationship was not significant for newly industrialized countries similar to present study conducted in Mathura, India [20]. This might be attributed to the fact that both obesity and dental caries are multifactorial in aetiology and various genetic and environmental factors have an impact on them. Another risk factor common to both obesity and dental caries is high sugar intake. Ludwig et al. [21], in a longitudinal study, found that the increasing prevalence of obesity in children was linked to the consumption of sugar-sweetened drinks. However, our study found no significant association between dental caries (DMFT/dmft) and daily sugar intake. Even with increased consumption or high intake of sugar there was decrease in dental caries. This might be attributed to the widespread exposure to fluoride not only through drinking water but also through toothpaste, professional applications, and through fluoride's presence in processed foods and drinks [11]. This result is consistent with the findings of systematic review by Burt and Pai [11] which concluded that the relationship between sugar consumption is much weaker in modern age of fluoride exposure. Another study by Loveren [22] concluded that if good oral hygiene is maintained and fluoride is supplied frequently, teeth will remain intact even if the carbohydrate-containing food is frequently eaten. Local oral factors such as retention around the teeth and salivary functions may be factors strongly modifying caries activity [22]. Oral hygiene is a basic factor for oral health. Poor oral hygiene leads to accumulation of dental plaque which has an important role in the aetiology of dental caries [23]. The overall oral hygiene status among study population was recorded as fair in 65% and good in 31% and only 4% of the study population showed poor oral hygiene status. There was significant difference between oral hygiene status of males and females (*P* = 0.037). The OHI-S and its components showed a high mean value for males as compared to females. The probable reason for lower mean scores of OHI-S and its components in females was perhaps the increased grooming habits of girls in this age group. These findings are in accordance with the study by Sogi and Bhaskar [24]. Even though oral hygiene status of majority of the study population was between fair and good, 27% of the study subjects were affected by caries in the present study but no statistically significant difference was seen between DMFT/dmft and oral hygiene status (DMFT, *P* = 0.082; dmft, *P* = 0.354). However, multiple linear regression analysis found that oral hygiene status had a significant effect on caries prevalence (OR = 5.061, *P* = 0.004, S).

## 5. Conclusion

Oral hygiene status had an intricate relationship with caries prevalence whereas body mass index and daily sugar intake did not reveal any significant association in 12-year-old school children of Mathura city. The relationship between dental caries and obesity should be further explored by longitudinal studies as they both have common risk determinants.

## Figures and Tables

**Table 1:** 

Form	Frequency	Points
Liquid: soft drinks, fruit drinks, cocoa, sugar and honey in beverages, nondairy creamers, ice cream, sherbet, gelatine desert, flavoured yoghurt, pudding, custard, popsicles	— × 5 =	



Solid and sticky: cake, cupcakes, donuts, sweet rolls, pastry, canned fruit in syrup, bananas, cookies, chocolate candy, caramel, toffee, jelly beans, other chewy candies, chewing gum, dried fruit, marshmallows, jelly, jam	— × 10 =	



Slowly dissolving: hard candies, breath mints, antacid tablets, cough drops	— × 15 =	

Total sweet score: —		
Interpretation sweet score: 5 or less: excellent 10: good 15 or more: “watch out” zone		

Total sweet score: —

Interpretation sweet score: 5 or less: excellent; 10: good; and 15 or more: “watch out” zone.

**Table 2:** Distribution of various characteristics of study population.

Sociodemographic characteristics	Study subjects	
	(*n* = 100)	
	Male (*n* = 50)	Female (*n* = 50)
Diet		
Vegetarian	41 (82%)	44 (88%)
Mixed	9 (18%)	6 (12%)
Oral hygiene means		
Toothbrush with toothpaste	45 (90%)	50 (100%)
Toothbrush with toothpowder	4 (8%)	0 (0%)
Indigenous (chewing stick)	1 (2%)	0 (0%)
Oral hygiene frequency		
Once	38 (76%)	31 (62%)
Twice	12 (24%)	19 (38%)
Body mass index categories		
Underweight**	4 (8%)	23 (46%)
Healthy weight**	40 (80%)	24 (48%)
At risk of overweight	5 (10%)	3 (6%)
Overweight**	1 (2%)	0 (0%)
Daily sugar intake		
Excellent	8 (16%)	3 (6%)
Good	12 (24%)	17 (34%)
Watch out	30 (60%)	30 (60%)
Oral hygiene status		
Good*	12 (24%)	19 (38%)
Fair	34 (68%)	31 (62%)
Poor*	4 (8%)	0 (%)

**represents that values obtained are highly statistically significant (*P* < 0.001).

*represents that values obtained are statistically significant (*P* < 0.05).

**Table 3:** Multiple linear regression analysis of oral hygiene status, body mass index, and daily sugar intake on caries prevalence in 12-year-old school children.

Independent variables	Dependent variable (caries affected at 12 years of age)				
	Odd ratio	95% CI		SE	*P* value
		Lower	Upper		
Body mass index	0.742	0.365	1.511	0.363	0.411
Daily sugar intake	1.214	0.613	2.407	0.349	0.578
Oral hygiene status	5.061	1.669	15.347	0.566	**0.004**

## References

[1] Reich E, Lussi A, Newbrun E (1999). Caries-risk assessment. *International Dental Journal*.

[2] Edelstein BL (2006). The dental caries pandemic and disparities problem. *BMC Oral Health*.

[3] Baehni PC, Guggenheim B (1996). Potential of diagnostic microbiology for treatment and prognosis of dental caries and periodontal diseases. *Critical Reviews in Oral Biology and Medicine*.

[4] http://www.whocollab.od.mah.se/countriesalphab.html.

[5] Dash JK, Sahoo PK, Bhuyan SK, Sahoo SK (2002). Prevalence of dental caries and treatment needs among children of Cuttack (Orissa). *Journal of the Indian Society of Pedodontics and Preventive Dentistry*.

[6] Pinto A, Kim S, Wadenya R, Rosenberg H (2007). Is there an association between weight and dental caries among pediatric patients in an urban dental school? A correlation study. *Journal of Dental Education*.

[7] http://en.wikipedia.org/wiki/Obesity_in_India.

[8] Sadeghi M, Alizadeh F (2007). Association between dental caries and body mass index-for-age among 6-11-year-old children in Isfahan in 2007. *Journal of Dental Research, Dental Clinics, Dental Prospects*.

[9] Kopycka-Kedzierawski DT, Auinger P, Billings RJ, Weitzman M (2008). Caries status and overweight in 2- to 18-year-old US children: Findings from national surveys. *Community Dentistry and Oral Epidemiology*.

[10] Gerdin EW, Angbratt M, Aronsson K, Eriksson E, Johansson I (2008). Dental caries and body mass index by socio-economic status in Swedish children. *Community Dentistry and Oral Epidemiology*.

[11] Burt BA, Pai S (2001). Sugar consumption and caries risk: a systematic review. *Journal of dental education*.

[12] Petti S, Tarsitani G, Panfili P, D’Arca AS (1997). Oral hygiene, sucrose consumption and dental caries prevalence in adolescent systemic fluoride non-users. *Community Dentistry and Oral Epidemiology*.

[13] Kuczmarski RJ, Ogden CL, Guo SS (2002). 2000 CDC Growth Charts for the United States: methods and development. *National Center for Health Statistics*.

[14] World Health Organization (1997). *Oral Health Surveys—Basic Methods*.

[15] Greene JC, Vermillion JR (1964). The simplified oral hygiene index. *Journal of the American Dental Association*.

[16] Darby ML, Walsh MM (2003). Nutritional counseling. *Dental Hygiene Theory and Practice*.

[17] David J, Wang NJ, Åstrøm AN, Kuriakose S (2005). Dental caries and associated factors in 12-year-old schoolchildren in Thiruvananthapuram, Kerala, India. *International Journal of Paediatric Dentistry*.

[18] Tramini P, Molinari N, Tentscher M, Demattei C, Schulte AG (2009). Association between caries experience and body mass index in 12-year-old French children. *Caries Research*.

[19] Kantovitz KR, Pascon FM, Rontani RMP, Gavião MBD (2006). Obesity and dental caries—a systematic review. *Oral Health & Preventive Dentistry*.

[20] Hayedn C, Bowler JO, Chambers S (2013). Obesity and Dental caries in children: a systematic review and meta-analysis. *Community Dentistry and Oral Epidemiology*.

[21] Ludwig DS, Peterson KE, Gortmaker SL (2001). Relation between consumption of sugar-sweetened drinks and childhood obesity: a prospective, observational analysis. *The Lancet*.

[22] Loveren C (2000). Diet and dental caries: cariogenicity may depend more on oral hygiene using fluorides than on diet or type of carbohydrates. *European Journal of Paediatric Dentistry*.

[23] http://www.whocollab.od.mah.se/expl/ohiintrod.html.

[24] Sogi G, Bhaskar DJ (2001). Dental caries and oral hygiene status of 13-14 year old school children of Davangere. *Journal of the Indian Society of Pedodontics and Preventive Dentistry*.

